# Lignin, mitochondrial family, and photorespiratory transporter classification as case studies in using co-expression, co-response, and protein locations to aid in identifying transport functions

**DOI:** 10.3389/fpls.2014.00075

**Published:** 2014-03-17

**Authors:** Takayuki Tohge, Alisdair R. Fernie

**Affiliations:** Department 1 (Willmitzer), Central Metabolism, Max Planck Institute for Plant PhysiologyPotsdam, Germany

**Keywords:** informatic identification, transporters, plant metabolism, co-expression network

## Abstract

Whole genome sequencing and the relative ease of transcript profiling have facilitated the collection and data warehousing of immense quantities of expression data. However, a substantial proportion of genes are not yet functionally annotated a problem which is particularly acute for transport proteins. In *Arabidopsis*, for example, only a minor fraction of the estimated 700 intracellular transporters have been identified at the molecular genetic level. Furthermore it is only within the last couple of years that critical genes such as those encoding the final transport step required for the long distance transport of sucrose and the first transporter of the core photorespiratory pathway have been identified. Here we will describe how transcriptional coordination between genes of known function and non-annotated genes allows the identification of putative transporters on the premise that such co-expressed genes tend to be functionally related. We will additionally extend this to include the expansion of this approach to include phenotypic information from other levels of cellular organization such as proteomic and metabolomic data and provide case studies wherein this approach has successfully been used to fill knowledge gaps in important metabolic pathways and physiological processes.

## INTRODUCTION

Over the last 15 years or so, co-expression analysis has emerged as a powerful statistical tool which is based on the guilt-by-association approach. This approach assumes that if transcript levels of a gene of unknown function correspond tightly with those of genes of known function then it is highly likely that the gene of unknown function plays a role in the same biological process as the known gene (Tohge and Fernie, 2012; Saito et al., 2013; Stitt, 2013). There are a number of caveats to this approach including the influence of the type of expression data used to construct the co-expression networks and the statistical methods used to evaluate them. However, these have been discussed in detail elsewhere [see reviews by (Usadel et al., 2009; Bordych et al., 2013; Stitt, 2013)] and when properly considered this strategy can prove very effective.

The earliest large scale use of this approach was performed in yeast in a two step approach. First, similarity scores were assigned to each possible gene pair on the comparison of their gene expression levels across a wide range of conditions. Secondly the resultant distance matrix, comprised of all possible similarity scores was organized (“clustered”), in a manner allowing the facile identification of genes showing the most similar expression patterns (Eisen et al., 1998). Given the simplicity of this approach it has also been rapidly adopted in microbial (Fribourg et al., 2001; Gasch and Eisen, 2002; Mao et al., 2005; Zhang et al., 2005), mammalian (Taniguchi et al., 2002; Voehringer et al., 2000; Altman and Raychaudhuri, 2001; Raychaudhuri et al., 2001; Lee et al., 2004; Li et al., 2004; Prieto et al., 2008), and plant research (Maleck et al., 2000; Schaffer et al., 2001; Goda et al., 2008). For plant research several web-based tools including ATTED-II (Obayashi et al., 2009; Obayashi et al., 2011), AraNet (Hwang et al., 2011), Expression Angler of the Bio-Array resource [BAR; (Toufighi et al., 2005], KappaViewer (Sakurai et al., 2011), GeneCAT (Mutwil et al., 2008), Genevestigator (Zimmermann et al., 2004), and VirtualPlant (Katari et al., 2010) simplify this task yet further. One of the strongest demonstrations of the power of this technique in plants comes from its early utility in the identification of further genes involved in secondary cell wall and hemicellulose synthesis in *Arabidopsis* (Brown et al., 2005; Persson et al., 2005; Cocuron et al., 2007). These studies used the three major cellulose synthase A (CESA) genes as a bait (i.e., expression profile query), to construct networks and thus isolate novel functional genes displaying similar expression patterns. Subsequent confirmation of the biological function of a large number of these genes has been achieved and documented in a number of publications (Bringmann et al., 2012; Ruprecht and Persson, 2012; Sanchez-Rodriguez et al., 2012). A second area of plant metabolism in which the approach has proven highly informative is secondary metabolism. This is perhaps not surprising since secondary metabolism is often regulated directly at the transcriptional level and known to be under the control of a wide range of transcription factors including the MYB transcription factors. The initial use of this approach was in constructing a flavonoid co-expression network to identify a flavonol-3′-*O*-methyltransferase (Tohge et al., 2007) and flavonol-7-*O*-rhamnosyltransferse (Yonekura-Sakakibara et al., 2007). However, it has subsequently been used to find other flavonoid genes (Yonekura-Sakakibara et al., 2008), glucosinolate MYB regulators (Hirai et al., 2007), anthocyanin glucosyltransferase (Yonekura-Sakakibara et al., 2012), phospholipid sugar transferase (Okazaki et al., 2009), and lignin biosynthetic genes (Ehlting et al., 2005; Vanholme et al., 2013). These studies have thus allowed us to make considerable advances in defining genes associated with metabolism *per se*. That said, even for primary metabolism, there remain many essential proteins for which the corresponding gene has not yet been identified. This is particularly problematic for transport proteins, of which estimates based on entirely different approaches suggest that a total of 6500 membrane transporters exist in *Arabidopsis* (Schwacke et al., 2003), while 700 intracellular transporters are required merely to maintain the primary metabolic network of the same species (Mintz-Oron et al., 2012). Recent articles concerning chloroplast-, peroxisomal-, vacuolar-, ER-, and plasma membrane-transport all indicate a gradual increase in the functional elucidation of all types of transport proteins (Liu and Bush, 2006; Palmieri et al., 2011; Rieder and Neuhaus, 2011; Weber and Linka, 2011; Martinoia et al., 2012; Hoffmann et al., 2013). However, the number of identified transporter proteins falls well short of either of the predicted numbers given above, i.e., there are a vast number of putative transport proteins but the vast majority have either only homology-based annotations or no functional characterization whatsoever. The recent review by Schroeder et al. (2013) reiterates the importance of transporters in metabolic engineering strategies and as such defines the identification of the permeome as of clear strategical importance in sustaining crop productivity. As one approach toward this goal, in this mini-review we detail (i) the potential of co-expression as a stand-alone approach for aiding in the definition of metabolite transporters and (ii) how other phenotypic data can be integrated with that of gene expression in order to enhance chances of successful gene annotation.

### IDENTIFICATION OF TRANSPORTERS IN THE MODEL PLANT *Arabidopsis*

As mentioned above bioinformatics strategies based either on features in protein amino acid sequences or on transport steps required to allow a functional subcellular metabolism have enable us to set an upper limit to the number of metabolite transporters in the plant cell. Despite this considerable research effort is warranted to elucidate the function of these carriers. That said, amongst many important breakthroughs in transporter identification, critical advances have been made both in the cloning of the first transporters of the core photorespiratory pathway (Bordych et al., 2013; Pick et al., 2013) and amino acid metabolism (Liu and Bush, 2006) as well as the initial characterization of a glucosinolate transporter (Gigolashvili et al., 2009; Sawada et al., 2009), epicatechin conjugates transporter (Marinova et al., 2007), and a lignol transporter (Alejandro et al., 2012). In this section we will detail the role of co-expression studies in these discoveries (**Table 1**).

**Table 1:** A table summarizing the transporter genes presented in this review.

Gene name		AGI	Network	Reference
PLGG1	Plastidal glycolate glycerate translocator 1	At1g32080	Photorespiratory metabolism	Pick etal. (2013)
BOU	A BOUT DE SOUFFLE	At5g46800	Photorespiratory metabolism	Eisenhut etal. (2013), Lawand etal. (2002)
AtDiT1	Plastidial 2-oxoglutarate/malate transporter	At5g12860	Photorespiratory metabolism	Taniguchi etal. (2002), Renné etal. (2003)
BAT5	2-keto acids transporter	At4g12030	Glucosinolate biosythesis	Gigolashvili etal. (2009), Sawada etal. (2009)
NTR/PTR	Glucosinolate transporter	At3g47960	Glucosinolate biosythesis	Nour-Eldin and Halkier (2013)
AtABC29/PDR1	Monolignol transporter	At3g16340	Lignin biosynthesis	Alejandro etal. (2012)

#### Transporters involved in photorespiration

The use of co-expression analysis with regard to the identification of photorespiratory transporters has recently been expertly reviewed (Bordych et al., 2013) so we will only cover it briefly here. In their analysis the gene PLGG1 (At1g32080) was ranked as a highly promising candidate transporter and plgg1-1 knockout plants develop chlorotic regions along the leaf lamina when grown under ambient air (NC) conditions while the transporter was recently characterized as the plastidic glycerate/glycolate transporter (Pick et al., 2013). Similarly, the A BOUT DE SOUFFLE (BOU) protein was successfully identified as a transporter involved in shuttling intermediates in the photorespiratory C2 cycle (Lawand et al., 2002; Eisenhut et al., 2013). *Bou* knockout plants were demonstrated to suffer in ambient air, but grow much like the wild-type when kept under high CO_2_ conditions. Moreover, the glycine level was greatly increased in comparison to that of wild-type plants, while mitochondrial glycine degradation is strongly reduced in the mutant. Although the specific substrate transported via BOU has not been identified, results collated to date seem to suggest it is likely to be a glycine decarboxylase co-factor (Bordych et al., 2013). A third candidate, the plastidial 2-oxoglutarate (2-OG)/malate transporter (AtDiT1) was found in a co-expression analysis approach and its sequence homology with DiT2.1 (AtpDCT1; Taniguchi et al., 2002; Renné et al., 2003). The function of this gene was subsequently confirmed by phenotypic analysis of the gene knockout mutant (*dit1* mutant) which was shown to suffer under normal growth conditions, and displayed retarded development, small leaf size, frequently emerging shoots, and a decrease in chlorophyll content (Kinoshita et al., 2011). AtDiT1 provides the chloroplast with the 2-OG utilized by Ferredoxin-dependent glutamate synthase (FD-GOGAT) in the chloroplast and constructs a double-transporter system together with the AtpDCT1 protein. Thus this protein participates, albeit one step removed, in the export of synthesized glutamate and re-fixation of ammonium ions as the result of the photorespiratory cycle (Schneidereit et al., 2006; Kinoshita et al., 2010). Despite the success of these three examples the function of the other genes highlighted in this photorespiratory co-expression study remain to be confirmed.

#### Bile acid transporter family

The plastidic bile acid transporter 5 (BAT5) was associated with glucosinolate metabolism on the basis of its co-expression with known genes of glucosinolate metabolism (Gigolashvili et al., 2009; Sawada et al., 2009). This was importantly confirmed by the fact that loss of function and reduced expression of BAT5 resulted in considerably decreased glucosinolate levels (Gigolashvili et al., 2009; Sawada et al., 2009). However, sodium-coupled transport activity of recombinant BAT5 has yet to be demonstrated. Recently, glucosinolate transport to seeds was characterized as being carried out by At3g47960, a member of the nitrate/peptide (NTR/PTR) transporter family, in an approach independent of co-expression analysis (Nour-Eldin and Halkier, 2013). Returning to the BAT, using a targeted variation on the co-expression theme, another member of this family – namely BAT1 – was putatively identified (and subsequently confirmed), as a plastidial sodium-dependent pyruvate transporter (Furumoto et al., 2011). In this study the authors used comparative transcriptome analyses between a C_3_ plant species, *Flaveria pringlei*, and the closely related C_4_ plant species *F. trinervia *and *F. bidentis* to identify three novel C_4_ species abundant genes predicted to encode chloroplast membrane proteins. Unlike C_3_ plant species, which only contain a sodium-dependent pyruvate transporter, both sodium-dependent and sodium-independent pyruvate transport have been reported in a range of C_4_ species (Aoki et al., 1992). Given this fact Furumoto et al. (2011) used their cross-species analyses to search for the gene encoding the sodium-dependent pyruvate transporter using the following criteria; (i) given its essential role in C_4_ photosynthesis it should be expressed at considerably higher levels in C_4_ than C_3_ plants and (ii) that its expression should be low in plants of the proton-dependent C_4_ plant species but equivalent in plants displaying sodium-dependent pyruvate transport. Wider comparative transcriptomics allowed the exclusion of one of the three candidate genes. Crucially functional analysis revealed BAT1, on the basis of its efficient import of pyruvate and physiological characterization of *Arabidopsis* mutant, to be the plastid sodium-dependent pyruvate carrier (Furumoto et al., 2011). In this study the authors were further able to pinpoint BAT1 as functioning in C_4_ and in the methyl erythritol phosphate pathway in C_3_ plants. The search for the mitochondrial pyruvate transporter is, however, ongoing.

#### Lignin transporters

In order to identify genes involved in monolignol transport, Alejandro et al. (2012) performed a co-expression network analysis with the ABCG transporter subfamily (previously called WBCs and PDRs) of *Arabidopsis* using the ATTED-II database (). Given that members of the ABCG subfamily have been shown to transport a broad range of fatty acids and terpenoids they wondered whether this class could also be implicated in the transport of phenolic compounds. The results revealed that AtABCG29/PDR1, a member of the full-size ABCG subfamily, exhibited a high co-expression ratio with three genes of the phenylpropanoid biosynthesis pathway, which is involved in the synthesis of lignin and flavonoids. The well-correlated genes correspond to two 4-coumarate coenzyme A (CoA) ligases (4CL2 and 4CL5), which convert hydroxycinnamic acids into hydroxycinnamoyl CoA esters, and one caffeoyl CoA-*O*-methyltransferase catalyzing the conversion of caffeoyl CoA into feruloyl CoA. Moreover, seven further genes related to phenylpropanoid biosynthesis are co-expressed with AtABCG29, albeit with lower co-expression ratios. In concordance with these results Ehlting et al. (2005) also reported that AtABCG29 showed an expression pattern in primary stems consistent with that of monolignol biosynthetic genes and increased lignin content. Subsequent characterization of AtABCG29 revealed that yeasts expressing this transporter exhibited increased tolerance to *p*-coumaryl by means of excreting this monolignol whilst AtABCG29 deficient mutants revealed that they contained less lignin as well as modifications to secondary metabolites underlining the importance of* p*-coumaryl alcohol levels in the cytosol (Alejandro et al., 2012). Similarly, a targeted co-expression analyses looking for transporters which were specifically highly expressed in the phloem was used alongside metabolome analyses to uncover that *ABCG9*, *ABCG11*, and *ABCG14 *are involved in lipid/sterol homeostasis regulation (Le Hir et al., 2013).

As stated above a vast number of transport proteins remain uncharacterized and physiologically important transporters such as the mitochondrial pyruvate and folate transporters as well as practically all amino acid transporters remain to be molecularly characterized. The examples presented here suggest that the co-expression approach will have utility in identifying genes encoding transporters for specific metabolites.

#### Use of co-expression analysis in pinpointing process-related transporters

With the exception of the identification of the photorespiratory transporters described above most uses of co-expression we have described thus far have related to the identification of (metabolite) specific transporters, however, the utility of the approach goes far beyond this application. The photorespiratory transporters are the best example to date of taking a broader approach, however, several further studies have followed this route albeit not to such a conclusive end. Three examples of this come from our own work wherein we looked at (i) genes co-expressed on dark induced senescence (Araújo et al., 2010; Araújo et al., 2011), (ii) genes co-expressed following exposure to high levels of several light species including UV-B irradiance (Tohge et al., 2011), and (iii) genes co-expressed with barley tonoplast proteins (Tohge et al., 2011). The first two approaches identified nine transporters and nine transport related proteins as putative membrane transporters involved in senescence and the UV-B responsive phenolic secondary metabolism, respectively. The former study exhibited considerable overlap in targets to co-expression and *cis* associated regulatory element analysis of mitochondrially associated proteins following imposition of a broad range of mitochondrial stresses (Holt et al., 2006), providing further support for the correctness of the putative functional assignment which we suggested. The latter study was, however, slightly more complicated in that it formed clusters on the basis of already identified tonoplast proteins but gave suggested functions including transport of phenylpropanoids (Multidrug resistant type transporter and H+ dependent transporter) and mugineic acid (ABC transporter and transport related protein which is in the gene family of glutathione *S*-transferase). It is important to note, however, that these candidate genes are yet to be validated by functional analysis.

## CONCLUSION AND OUTLOOK

Recent years have seen impressive advances in our understanding of transport protein function, however, many gaps remain (Weber and Linka, 2011; Rolland et al., 2012; Sweetlove and Fernie, 2013). While the co-expression approach has been used effectively for transport function predictions being, at least partially, responsible for many, of the discoveries reviewed in this article it probably remains an underexploited tool. The layering of datasets beyond those at the transcriptional level (Tohge and Fernie, 2012), alongside more sophisticated cross-species comparisons such as that illustrated in the Furomoto study will ultimately likely be more tractable in asking specific pathway or process based questions. That said the recent characterization of the plant ammonium transceptor De Michele et al. (2013) suggests, at least in theory, that such approaches may ultimately also prove powerful in linking transporters to signal transduction cascades and the processes which they control.

## Conflict of Interest Statement

The authors declare that the research was conducted in the absence of any commercial or financial relationships that could be construed as a potential conflict of interest.
